# Implementation strategies of internet-based asthma self-management support in usual care. Study protocol for the IMPASSE cluster randomized trial

**DOI:** 10.1186/1748-5908-7-113

**Published:** 2012-11-21

**Authors:** Johanna L van Gaalen, Moira J Bakker, Leti van Bodegom-Vos, Jiska B Snoeck-Stroband, Willem JJ Assendelft, Ad A Kaptein, Victor van der Meer, Christian Taube, Bart P Thoonen, Jacob K Sont

**Affiliations:** 1 Department of Medical Decision Making, Leiden University Medical Centre, P.O. Box 9600, 2300, RC, Leiden, the Netherlands; 2 Department of Public health and Primary care, Leiden University Medical Centre, P.O. Box, 9600, 2300, RC, Leiden, the Netherlands; 3 Department of Medical psychology, Leiden University Medical Centre, P.O. Box, 9600, 2300, RC, Leiden, the Netherlands; 4 Department of Pulmonology, Leiden University Medical Centre, P.O. Box, 9600, 2300, RC, Leiden, the Netherlands; 5 Department of General Practice, Radboud University Nijmegen Medical Centre, P.O. Box 9101, 6500, HB, Nijmegen, the Netherlands

**Keywords:** Asthma, Self-management, Telemanagement, E-health, Self-management, Implementation, Chronic care

## Abstract

**Background:**

Internet-based self-management (IBSM) support cost-effectively improves asthma control, asthma related quality of life, number of symptom-free days, and lung function in patients with mild to moderate persistent asthma. The current challenge is to implement IBSM in clinical practice.

**Methods/design:**

This study is a three-arm cluster randomized trial with a cluster pre-randomisation design and 12 months follow-up per practice comparing the following three IBSM implementation strategies: minimum strategy (MS): dissemination of the IBSM program; intermediate strategy (IS): MS + start-up support for professionals (*i.e.*, support in selection of the appropriate population and training of professionals); and extended strategy (ES): IS + additional training and ongoing support for professionals. Because the implementation strategies (interventions) are primarily targeted at general practices, randomisation will occur at practice level.

In this study, we aim to evaluate 14 primary care practices per strategy in the Leiden-The Hague region, involving 140 patients per arm. Patients aged 18 to 50 years, with a physician diagnosis of asthma, prescription of inhaled corticosteroids, and/or montelukast for ≥3 months in the previous year are eligible to participate. Primary outcome measures are the proportion of referred patients that participate in IBSM, and the proportion of patients that have clinically relevant improvement in the asthma-related quality of life. The secondary effect measures are clinical outcomes (asthma control, lung function, usage of airway treatment, and presence of exacerbations); self-management related outcomes (health education impact*,* medication adherence, and illness perceptions); and patient utilities. Process measures are the proportion of practices that participate in IBSM and adherence of professionals to implementation strategies. Cost-effective measurements are medical costs and healthcare consumption. Follow-up is six months per patient.

**Discussion:**

This study provides insight in the amount of support that is required by general practices for cost-effective implementation of IBSM. Additionally, design and results can be beneficial for implementation of other self-management initiatives in clinical practice.

**Trial registration:**

the Netherlands National Trial Register NTR2970

## Background

Asthma is a common chronic inflammatory disease of the airways, typically characterized by symptoms such as wheeze, shortness of breath, and coughing [1]. Despite the wide availability of effective therapy, long-term management of asthma falls for short of the goals set in guidelines [2], and many patients do experience a profound burden of disease [3].

Self-management is an essential component in the proactive management of asthma [1,4] because it helps patients to reach their treatment goals and enables patients to manage symptoms, treatment, physical and psychosocial consequences, and lifestyle changes inherent in living with a chronic condition [5]. However, the uptake of self-management in clinical practice may be hampered because easy-use tools that enhance sustained uptake of action plan usage by patients are lacking in today’s practice [6], and patients can experience a lack of ownership of these action plans [7]. Not surprisingly, a minority of general practices provide patients with written action plans [8,9].

Internet technology is increasingly being seen as an appealing tool for self-management for patients with chronic disease [10-12]. Telehealth care in asthma is defined as healthcare being delivered from a distance, facilitated electronically, and involving the exchange of information through the personalized interaction between a healthcare professional using their skills, judgment, and the patient providing information [13]. Telehealth care may overcome barriers towards optimal care in patients with mild to moderate asthma [14]. More specifically, internet technology can be employed for ongoing individualized management of the patient [15].

Internet-based self-management (IBSM) support in asthma consists of the following components: internet-based asthma monitoring, internet-based goal setting, decision support with a treatment plan, online medical review, tailored online information, and communication with a healthcare provider. Recently, we have shown that such IBSM can improve asthma-related quality of life, asthma control, the number of symptom-free days, and lung function in patients with mild to moderate persistent asthma, as compared to usual care [16]. In a cost-utility analysis [17], it was demonstrated that IBSM support can be as effective as current asthma care with regard to quality of life in terms of patient utilities, and costs are similar over a one-year period.

Therefore, the current challenge is to implement IBSM support in routine asthma management within primary care. Patients that are most likely to be willing to participate and benefit from (internet-based) self-management are those with partially controlled or uncontrolled asthma [18-20]. These patients constitute about two-thirds of the asthma population in primary care [8].

A structured implementation strategy is needed to incorporate IBSM in current clinical practice and subsequently into a patient’s daily life. Implementation strategies for IBSM, consisting of several components (so-called ‘multi-faceted implementation strategy’) are suggested to be more effective in changing current clinical practices [21]. In addition, tailoring the implementation strategy to barriers and facilitators experienced by the target group—patients with asthma, practice nurses (PNs), and general practitioners (GPs)—is recommended [22-24]. Such barriers can be identified at different levels of healthcare system [25]: innovation, the individual patient (*i.e.*, illness perceptions), professional level, societal context (opinion of colleagues), organisational context (organisation of care process), and economic and political contexts.

Prior to this project, we conducted focus groups and interviews with patients and professionals for exploring barriers and facilitators for usage of IBSM in primary care [26]. These barriers were identified at patient and professional/organizational level. Main barriers at the patient level were unawareness of their level of asthma control and subsequent possibility for improvement, and patients often do not perceive asthma as a chronic condition and experience difficulties of integrating self-management activities into daily life. Main barriers at the professional level (PN, GP) and organizational level were unawareness of the level of asthma control of patients, lack of structure of asthma care, and lack of structure of routine asthma consultations within general practice and lack of time. Consequently, we developed three implementation strategies (the strategies will be described in more detail below):

1. Minimum strategy (MS): dissemination of the IBSM program.

2. Intermediate strategy (IS): MS + start-up support for professionals (*i.e.*, support in selection of the appropriate population and training of professionals).

3. Extended strategy (ES): IS + additional training and ongoing support for professionals.

In summary, the MS strategy has not been tailored to previously identified barriers and corresponds with commonly used implementation strategies (*i.e.*, dissemination of the innovation only). This is in contrast with the IS strategy, which specifically have been developed for addressing previous identified barriers. The ES strategy is the most extensive and time-intensive strategy. Currently, there are only sparse data on the effectiveness and cost-effectiveness of implementation strategies for IBSM in primary care. This information is particularly important for the time-intensive implementation interventions, such as selection of the appropriate population, professional training, and ongoing support for professionals in IBSM support.

### Hypotheses

To evaluate the impact of these three different implementation strategies for IBSM in current clinical practice, we have proposed four hypotheses, which are constructed to compare the effect of tailoring implementation strategies to identified barriers (IS and ES) versus a commonly used, non-tailored strategy (MS):

1. More general practices will participate in IBSM in the IS or ES strategy as compared with the MS strategy;

2. The proportion of referred patients who participate in the IBSM program in the ES or IS strategy will be greater as compared with the MS strategy;

3. The proportion of referred patients who participate in the IBSM program in the ES or IS strategy will be greater as compared to the MS strategy;

4. The ES and the IS strategy will be more cost-effective as compared to the MS strategy.

### Objectives

The objectives of this study are to investigate the effectiveness and cost-effectiveness of a MS strategy, as compared to an IS strategy and an ES strategy in a three-arm, cluster randomized trial. Because these different implementation strategies have a sequence of effects, the evaluation is aimed to assess to what extent: practices participate in IBSM; IBSM improves asthma related quality of life; patients participate in IBSM; and the various implementation strategies are cost-effective.

## Methods

### Study design

**Figure 1:**
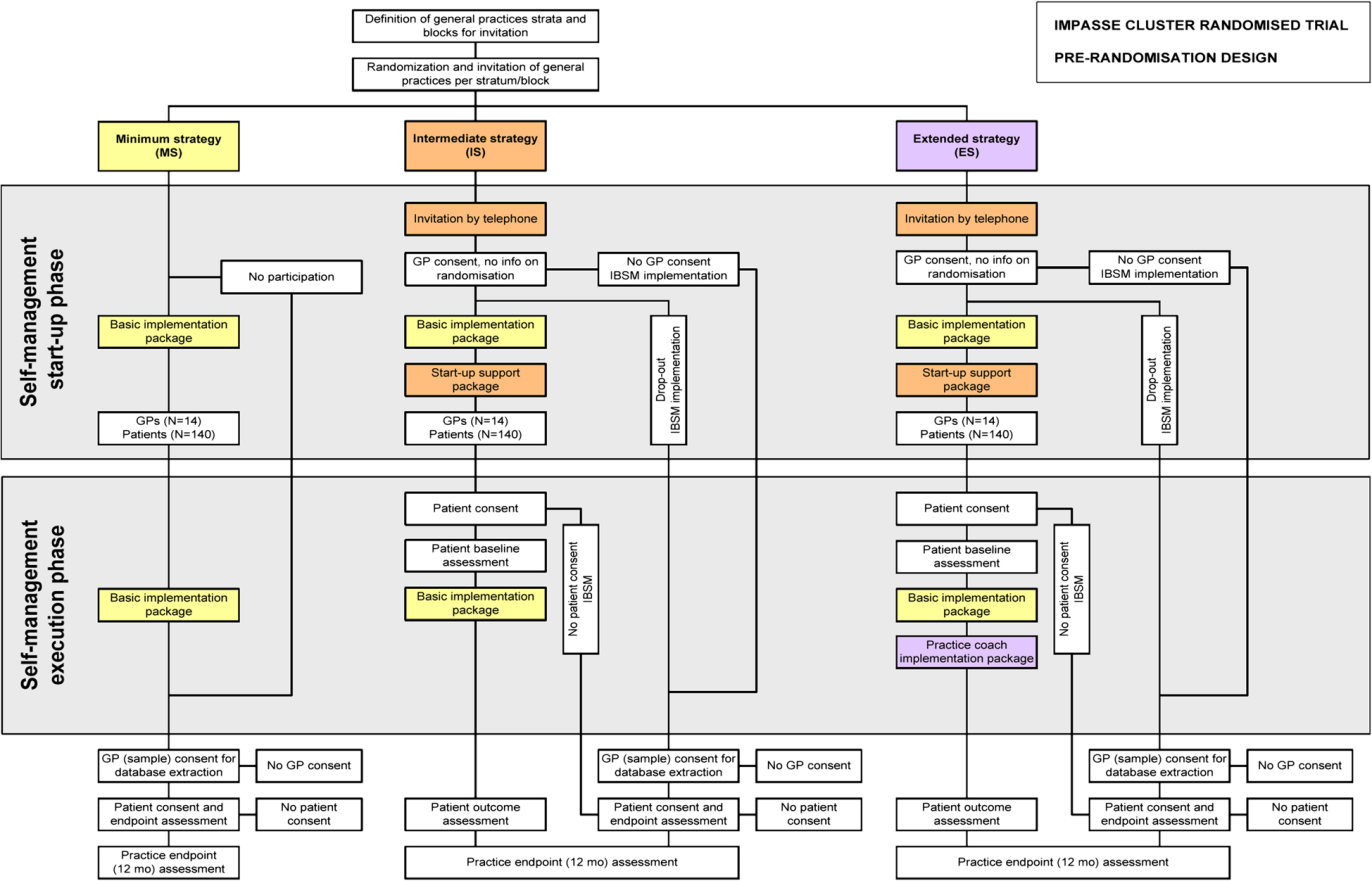
Study design.

This study is a three-arm, cluster randomized trial with a cluster pre-randomisation design [7] (Figure 1). Because the implementation strategies are primarily targeted at general practices, randomisation will occur at practice level (CONSORT guidelines for cluster trials, Table 1[27]). Prior to obtaining informed consent from GPs and patients, practices will be allocated to one of the strategies. Follow-up per practice is 12 months. At patient level, follow-up duration is six months. In the ES and IS strategies, individual patient outcomes will be evaluated at baseline (first visit of a patient to the general practice for instruction on IBSM), and three and six months after a patient’s start with IBSM. Individual patient outcomes in the MS strategy will be evaluated at six months (end-point evaluation) after a patient’s start with IBSM.

**Table 1:** **Consort checklist**[27]

**Item**	**Standard Checklist item**	
**Title**	Identification of study as randomised	Implementation strategies of internet-based asthma self-management support in usual care. Study protocol of the IMPASSE study - a cluster randomized trial
**Trial design**	Description of the trial design (e.g., parallel, cluster, non-inferiority)	Cluster-randomized trial with a cluster pre-randomization design.
**Methods**		
**Participants**	Eligibility criteria for participants and the settings where the data were collected	Eligibility criteria for general practices:
		Location within the Leiden - the Hague region. General practitioner/practice nurse that is willing and available to support patients in internet-based self-management.
		Patient eligibility criteria: Age 18 to 50 years; a doctor diagnosis of asthma; prescription of inhaled corticosteroids and/or montelukast ≥ three months within 12 months prior to enrolment; internet access; ability to understand written and oral Dutch instructions. Patient exclusion criteria: Severe co-morbidities, daily or alternate day oral corticosteroid therapy for ≥1 month prior to entering the study and being primarily under treatment by a respiratory physician.
		Data will be collected in a research module of the internet-based self-management support program (PatientCoach.nl) using web-based questionnaires (SurveyGizmo, Boulder, Colorado).
**Interventions**	Interventions intended for each group	Internet-Based Self-Management (IBSM) support program :
		PatientCoach.nl consists of both a generic web-based system and an instruction visit for patients. PatientCoach includes modules for self-monitoring (asthma control and lung function), a treatment plan (medication,), motivational feedback, e-consultation, personalized information (i.e., inhalation technique), reminders and forums for patients and professionals. PatientCoach has been developed by the LUMC. Patient Coach will be integrated in the general practice information system. Additionally PatientCoach contains a research module which consists of electronic versions of questionnaires.
		General practice level
		The implementation strategies for internet-based self-management support are primarily targeted general practices:
		1. Minimum strategy (MS): dissemination of the IBSM program.
		2. Intermediate strategy (IS): MS + start-up support for professionals (i.e., support in selection of the appropriate population and training of professionals).
		3. Extended strategy (ES): IS + additional training and ongoing support for professionals.
		All general practices will be asked to invite at least 10 patients to participate in PatientCoach. Follow-up duration at general practice level is one year.
		Patient level:
		Instruction visit on how to use PatientCoach, particularly focusing the essential self-management skills in asthma (i.e., monitoring, inhalation technique). Patients will be instructed to monitor their level of asthma control at least once per month, preferably once weekly using the Asthma Control Questionnaire. General practices themselves can decide whether the practice nurse and/or general practitioner guide patients in using PatientCoach. Follow-up per patient is six months. However, patients will have the possibility to continue using PatientCoach.nl after this period.
**Objective**	Specific objective or hypothesis	The objectives of this study are to investigate the effectiveness and cost- effectiveness of a Minimum strategy, as compared to an Intermediate strategy and an Extended strategy in a three arm cluster-randomized trial. Since these different implementation strategies have a sequence of effects, the evaluation is aimed to assess to what extent: 1. practices participate in IBSM (practice level); 2. IBSM improves asthma related quality of life (patient level); 3. patients participate in IBSM (patient level); and 4. The various implementation strategies are cost-effective (societal/organisational level)
**Outcome**	Clearly defined primary outcome for this report	Primary outcome measures are a) the proportion of referred patients that participate in IBSM (general practice (cluster) and patient level) and b) the proportion of patients that have clinically relevant improvement in the asthma-related quality of life as measured by the Asthma Quality of Life Questionnaire [30].Patient usage of IBSM is defined as two out of three months ACQ-monitoring compliance.
**Randomisation**	How participants were allocated to interventions	As the implementation strategies (interventions) are primarily targeted at general practices, randomisation will occur at practice level.
		General practices (i.e., 2,300 patients per ‘standard practice’) are the unit of randomisation. Prior to informed consent all general practices in the Leiden-the Hague region will be allocated to one of the three strategies (1:1:1 ratio). General practices receive a letter with information on the allocated strategy and an invitation to participate in the project.
		Randomization will be conducted by Jacob Sont using a computer-generated, permuted-block scheme. Practices will be stratified according the following characteristics: postal code (area) and practice size (practices with < 4 general practitioners are defined as a small practice, practices with ≥4 general practitioners as a large practice.
		General practices will be enrolled by Moira Bakker, Johanna van Gaalen and Jiska Snoeck-Stroband. Patients will be enrolled by general practices.
**Blinding (masking)**	**Whether or not participants, care givers, and those assessing the outcomes were blinded to group assignment**	All general practices and patients are blinded to group allocation. Researchers are not blinded for group allocation.
**Results**		
**Numbers randomized**	Number of participants randomized to each group	For all three strategies, 12 general practices (clusters) will be recruited, involving 10 patients per practice to be invited for using PatientCoach per general practice.
**Recruitment**	Trial status	Recruitment of patients is ongoing
**Numbers analysed**	Number of participants analysed in each group	Not applicable.
**Outcome**	For the primary outcome, a result for each group and the estimated effect size and its precision	Not applicable.
**Harms**	Important adverse events or side effects	Not applicable.
**Conclusions**	General interpretation of the results	This study provides insight in the amount of support that is required by general practices for cost-effective implementation of IBSM. Additionally, design and results can be beneficial for implementation of other self-management initiatives in clinical practice.
**Trial registration**	Registration number and name of trial register	the Netherlands National Trial Register NTR2970
**Funding**	Source of funding	This study is supported by grants from: - The Netherlands Organisation for Health Research and Development (ZON-MW 80-82315-97-10004) - The Netherlands Asthma Foundation (NAF 3.4.09.011) - Funding for this publication was obtained from the Netherlands Organisation for Scientific Research (NWO) Incentive fund Open Access publications - - Hand-held electronic lung function meters for patients (PikO-1, Ferraris Respiratory, Hertford, United Kingdom) were provided by GlaxoSmithKline (GSK), Zeist, the Netherlands

### Recruitment of general practices and patients

#### Eligibility criteria general practices

All general practices located within the Leiden – the Hague region and a GP/PN that is willing and available to support patients in IBSM will be eligible. Additionally, at least one GP per practice needs to give consent for participation.

#### Patients

General practices will be asked to invite at least ten 10 patients per practice to participate in IBSM. Based on previous studies on asthma within general practice, we know this is feasible [16]. Those patients not willing to participate in IBSM will be asked informed consent to participate in an endpoint evaluation at six months. Informed consent will be obtained during a consultation with a patient’s PN or GP.

#### Endpoint evaluation

Patients in practices randomized to the MS strategy, and those patients in the IS and ES strategy not willing to participate in IBSM support, will only be approached for an endpoint evaluation at six months after their start with PatientCoach.

#### Eligibility criteria patients

Patients, age 18 to 50 years, with a doctor diagnosis of asthma and prescription of inhaled corticosteroids and/or montelukast for at least three months in the previous year who have access to the internet are eligible to participate.

#### Exclusion criteria patients

Those who have severe co-morbidities (*i.e.*, terminal illness or a severe psychiatric disease), daily or alternate day oral corticosteroid therapy for at least a month before entering the study, or who are primarily under treatment by a respiratory physician are not eligible. Furthermore, the IBSM support program is not suitable for those who are unable to understand written and oral Dutch instructions.

#### Blinding and strategy allocation

General practices (*i.e.*, 2,300 patients per ‘standard practice’) will be the unit of randomisation. Practices will be stratified according the following characteristics: postal code (area) and practice size (practices with <4 GPs are defined as a small practice, practices with ≥4 GPs as a large practice). Prior to informed consent, practices will be randomized into one of the strategies (1:1:1 ratio), in order to assess the participation level of practices per strategy. Practices will receive a letter containing information on the allocated implementation strategy and an invitation to participate in the project.

Both practices and patients will be blinded to group allocation. Researchers will not be blinded for group allocation. Randomisation will be conducted by Jacob Sont using a computer-generated, permuted-block scheme. General practices will be invited until enough practices per strategy are participating. General practices will be enrolled by Moira Bakker, Johanna van Gaalen, and Jiska Snoeck-Stroband. Patients will be enrolled by general practices.

#### Sample size calculation

The sample size is based on patient participation in IBSM (primary outcome). We assume that one-third of the 15% to 20% of patients who have a written action plan [9] are actually using it to conduct self-management activities. Assuming that with the MS, 5% of the patients are frequently using the IBSM to monitor their asthma, we will consider the IS and ES successful if an increase of 25% is achieved (alpha 0.05 and beta 0.20). Using a correction for clustering of patients in practice (intra-cluster correlation coefficient: 0.25), we calculated that we need 10 patients in each of 42 practices 14 practices in the MS, IS, and ES, respectively to be invited to participate in IBSM using PatientCoach. This gives a total of 420 patients.

#### IBSM support program

The IBSM support program consists of both a generic web-based system and an instruction visit for patients.

#### PatientCoach

Patientcoach.nl is a generic web-based system that supports self-management of patients with a chronic condition. It includes modules for coaching, personalized information (*i.e.*, inhalation technique), self-monitoring, reminders, treatment plan, (motivational) feedback, e-consultations and a forum. PatientCoach has been developed by the Leiden University Medical Centre (LUMC) [16]. The program includes options for weekly assessment of the level of asthma control [28,29] and a quarterly-assessment of asthma-related quality of life [30]. Furthermore, it offers tools for professionals and patients, to help them to incorporate IBSM respectively into routine asthma care and daily life, such as: reminder options for home monitoring by ACQ and lung function; reminder options for routine consultations (*i.e.*, digital agenda); reminder options (*i.e.*, a general agenda) for regional educative sessions on asthma, *i.e.*, hosted by the patient association of the Netherlands Asthma association; and a forum for professionals.

PatientCoach contains a research module that consists of electronic versions of questionnaires for assessment of quality of life [31], health education impact [31], self-reported medication adherence [32], illness perceptions [33] and costs [34].

#### Self-management support session for patients on PatientCoach

Patients participating in PatientCoach will be supported by their PN and/or GP. PNs will be asked to invite the patient for at least one consultation (double-consultation) that aims to inform patients on how to use PatientCoach, particularly focusing the essential self-management skills in asthma (*i.e.*, monitoring, inhalation technique).

#### Usual care

PNs in all strategies will be asked to conduct follow-up on patients in concordance with the Dutch guideline for general practice on asthma in adults, which recommends a medical review and treatment adjustment every two to four weeks in unstable asthma and medical review once or twice a year for patients whose asthma is not under control [35]. The guideline states that routine asthma consultations include assessment of asthma control, medication, adverse events, adherence, and measurement of lung function. The guideline is concordant with current international guidelines, such as the Global Initiative for Asthma guideline [1].

#### Implementation strategies

The implementation period is divided in a start-up and an execution phase (Tables 2 and 3).

**Table 2:** Overview of implementation strategies

	Implementation strategy*		
	**MS**	**IS**	**ES**
**Start-up phase**			
**Practice recruitment**			
Recruitment letter	X	X	X
Reminder (letter)	X	X	X
Reminder (telephone)		X	X
**PatientCoach information**			
Manual	X	X	X
Information session	X	X	X
On site instruction		X	X
Workshop professionals	X	X	X
**Patient recruitment**			
By general practice	X	X	X
On site support (patient selection/invitation)	X	X	X
**PatientCoach tools**			
Reminders	X	X	X
**Execution phase**			
**Continuing support**			
Follow-up by the practice coach team			X
Outreach visit (if necessary)			X
Workshop professionals (implementation issues)			X
**Helpdesk**			
Technical issues (web-based/phone)	X	X	X
IBSM related issues (phone)	X	X	X

* MS = Minimum strategy; IS = Intermediate strategy; ES = extended strategy; IBSM = internet-based self-management support program (PatientCoach).

**Table 3:** Study phases and time schedule

	**Planning (months)**
Start-up phase	6
- General practice recruitment/Instruction general practices	6
- Patient recruitment	
Execution phase	6
- Patient follow-up	
Follow-up per general practice (includes both start-up and execution phase period)	12

All strategies consist of patient-directed (*i.e.*, reminders for consultations), professional-directed (*i.e.*, training of professionals, support on patient selection), and organisational components (*i.e.*, helpdesk).

### Minimum strategy (MS)

#### Start-up phase basic components

Practice recruitment: practices receive an information letter on IBSM support by using PatientCoach. Practices will receive a reminder letter within four weeks.

Participating physicians will be asked whether their non-responding colleagues in their practices would be interested in participation. On meetings for professional groups and patient organisations PatientCoach team members will present information on self-management and web-based support.

Patient recruitment: GPs are asked to invite at least ten patients to use PatientCoach.

PatientCoach information session for participating practices: In addition to a general announcement, professionals receive a manual on PatientCoach. Professionals (GP and/or PN) have the opportunity to join a PatientCoach information session. This information session is focused on the principles and usage of the IBSM program by the PN and patients, including home measurement of lung function as forced expiratory volume in one second (FEV_1_) using an electronic hand-held spirometer (PiKo-1: Ferraris; Hertford, UK). Finally, a short on-site training in usage of the PatientCoach system focusing on integration within the local primary care information system is offered to practices.

#### Execution phase basic components

Practices have access to a web-based helpdesk for technical issues. For issues related to IBSM in asthma, professionals can consult the PatientCoach team by email and/or telephone.

#### Intermediate strategy (IS)

This includes a start-up support package that is additional to the components of the MS in the start-up phase. During the execution phase the IS does not differ from the MS.

#### Start-up support package

The start-up support intermediate package consists of: Practice recruitment. General practices receive an invitation letter that includes all components of the Start-up Support Implementation strategy. Non-responding practices receive a reminder letter within four weeks and if necessary, practices are phoned.

On-site support for patient recruitment. PatientCoach team members offer practices the opportunity to make a patient selection in the patient registries of participating GPs.

PatientCoach start-up training. PNs and GPs are invited for an interactive session (duration: two hours) with colleagues. In this session, professionals are stimulated to discuss with each other on how to apply IBSM in their own practice. This training is focused on a proactive attitude toward patients and supporting them in their self-management skills by using IBSM support:

1. Exploration of a patient’s knowledge about asthma, illness perceptions [36,37], and the ability to integrate (internet-based) self-management activities into their daily life; *i.e.*, by exploring difficulties and discussing solutions.

2. Asthma control: assessment of the level of asthma control; patients are invited to discuss their most recent ACQ-score. Reasons for poor asthma control in patients are discussed, and the subsequent management strategy will be discussed.

3. Medication: information on side-effects; instruction, exploration of therapy adherence, and checking correct inhaler technique.

4. Asthma related lifestyle: smoking cessation, avoidance of triggers, regular exercise;

5. Asking about and treating rhinitis, which is known to influences asthma outcomes [38].

#### Extended strategy (ES)

The ES does not differ from the IS during the start-up phase. However, this strategy includes a patient coach package that is additional to the components of the MS/IS during the execution phase.

### Execution phase

#### Practice coach package

The practice coach package consists of the following components:Ongoing support: follow-up by PatientCoach team members at one, three, and five months after baseline by email, telephone, or face-to-face. During these planned consultations, members of the PatientCoach team will explore whether practices experience difficulties. Solutions and future recommendations are given. An outreach visit [23,39], both PatientCoach team initiated (planned) or professional initiated (unplanned), will be conducted when necessary.

Interactive session with professionals: within three months after starting to use PatientCoach, professionals will have the opportunity to participate in an interactive session with colleagues. This session will be focused on identifying problems for PatientCoach implementation, and solutions will be discussed.

#### Measurements and outcomes

Primary outcome measures are the proportion of referred patients that participate in IBSM and the proportion of patients that have clinically relevant improvement in the asthma-related quality of life as measured by the Asthma Quality of Life Questionnaire [30]. Patient usage of IBSM is based on monitoring of the level of asthma control using the Asthma Control Questionnaire [28,29]; monitoring of ACQ during two out of three months ACQ-monitoring compliance.

#### Patient level

Demographic characteristics (*i.e.*, educational level, atopy, smoking status, and symptom-free days) are obtained at baseline (Table 4). The effect evaluation includes clinical outcomes (asthma control, lung function, usage of airway treatment, and the presence of exacerbations) and self-management related outcomes (health education impact, self-reported medication adherence and illness perceptions) and patient utilities (asthma symptom utilities).

**Table 4:** Outcome measures

	**0 Months**	**3 Months**	**6 months / end-point**
**Demographic characteristics**	A		N
**Clinical**			
Asthma control (ACQ)	A	A,N	
Lung function (FEV_1_) []	A	A	A
**Quality of life/patient utilities**			
Asthma related quality of life (AQLQ)	A	A	A,N
Utilities: (EQ-5D)	X	X	A,N
**Self-management**			
Education (HeiQ)	X		X, N
Medication adherence (MARS)	X	X	X
Illness perceptions (B-IPQ)	X		X
**Costs**			
Healthcare and other costs (CostQ)	X	X	X, N

A = all strategies; X = Intermediate and Extended strategy; N = non-participating practices.

Asthma control is using the 7-item Asthma Control Questionnaire (ACQ), the optimal cut-point for ‘controlled’ is ≤0.75 and a value of ≥1.50 confirms ‘not controlled’ asthma [28]. Lung function will be measured as the Forced Expiratory Volume in one second (FEV_1_). Patients receive a handheld electronic spirometer (PiKo-1: Ferraris; Hertford, UK) and are instructed to report the highest value of three measurements in the morning before taking medication [1]. The presence of exacerbations is assessed; they are defined as deterioration in asthma that required emergency treatment or hospitalization (collected by quarterly questionnaire) or the need for oral steroids for three days or more (collected by pharmacy records) or the need for oral steroids for asthma as judged by the attending physician.

Exacerbations are defined according to ATS/ERS statement [40]:

Severe exacerbations require at least one of the following: systemic corticosteroids (tablets, suspension, or injection) usage or an increase from a stable maintenance dose, for three or more days; or a hospitalization or emergency room visit because requiring systemic corticosteroids.

Moderate exacerbations require ≥ 1 of the following features, lasting for two or more days: deterioration in symptoms, deterioration in lung function, and/or increased rescue bronchodilator use.”

Health education impact is assessed by the health education impact questionnaire (heiQ) [41]. Self-reported medication adherence is measured by the Medication Adherence Report Scale [32]. Illness perceptions are assessed by using the Brief Illness Perception Questionnaire (Brief IPQ) [33]. In addition, asthma symptom utilities are obtained from the Asthma Quality of Life Questionaire (AQLQ) [42], EQ-5D [31,43] and a visual analogue scale (VAS).

#### Professional and organizational level

Characteristics of general practices are assessed, including information on area, type of practice, years of establishment, age of GPs, and structure of asthma care. PN characteristics include gender, age, education, and years of experience with asthma consultations. The process evaluation contains outcomes on the adherence of professionals to the implementation strategy and can be considered as a feasibility evaluation. Attendance to training sessions of professionals is registered. Active referral of patients by professionals to PatientCoach is registered as active participation of practices. Actual treatment advice by the care provider and the treatment advice by the system are registered. Furthermore, frequency and time of IBSM usage by care providers are digitally logged.

#### Economic evaluation

The economic evaluation includes outcomes on cost-effectiveness and cost-utility. Medical costs, such as prescribed medication are assessed from electronic patient records (with written patient permission) complemented with the patient’s report on medication purchased elsewhere [44]. In addition, actual treatment advice by the care provider and treatment advice by the system are registered. Healthcare consumption, absenteeism and productivity loss, and the number of limited activity days are measured by using the Cost Questionnaire [34]. Furthermore, data on patient contacts (frequency of in-practice routine asthma consultations, telephone/email consultations, and unscheduled visits) are registered.

#### Analysis

The analysis is carried out on an intention-to-treat basis*.* Analyses include descriptive statistics and comparisons on process and effect evaluation. Differences are compared between implementation strategies using a random-effects analysis accounting for within-patient repeated measurements and clustering on general practice, or nonparametric comparisons, such as chi-square, as appropriate. All data are analyzed using STATA.

#### Analysis of clinical data

The number of patients with a clinically relevant improvement in asthma-related quality of life is based on a minimal clinical importance of difference (MCID) of 0.5 for the AQLQ [30].Patient utilities: asthma symptom utilities are obtained from the AQLQ [42]; indirect utilities from the general public are obtained using the EQ-5D [31,43]. This allows the calculation of quality-adjusted life years (Qalys). In the base case analysis, Qalys are estimated using societal utilities obtained using the Dutch EQ-5D tariff [45]. For sensitivity analyses, Qalys are estimated using a VAS (transformed using a power transformation). Data on healthcare provider utilization and other related costs provide input for calculation of the contract price that GPs will have to negotiate for this improved service. Ideally, this is in the form of a primary care diagnosis and treatment standard fee.

#### Economic evaluation

The economic evaluation compares differences in societal costs to differences in the number of limited activity days (CEA) and quality-adjusted life years (CUA). The analysis has a six-month time horizon, without discounting. Group averages are statistically compared using two-sided bootstrapping and net-benefit analyses are used to compare costs to patient outcome. Sensitivity analyses are performed on the perspective (societal versus healthcare perspective) and the applied utility measure (Dutch EQ5D, VAS).

#### Cost-effectiveness

Cost-effectiveness of treatment strategies is evaluated by incremental net-benefit analysis [46]. Net health benefit addresses cost-effectiveness ratios by assuming values for the willingness to pay per unit of effectiveness. The cost analysis includes both medical (medication, visits, and hospitalizations) and non-medical costs (productivity losses, informal care). Other costs are estimated using quarterly cost questionnaires [34] Costs are valued according to standard prices charges [47] including time and travel costs.

## Discussion

This study is designed to investigate the effectiveness and cost effectiveness of implementing IBSM by comparing three different implementation strategies (MS, IS, and ES) in a three-arm cluster-randomized trial with a cluster pre-randomization design. The IBSM application contains functionalities that are characterized by some innovative aspects, such as options for e-consultation and integration of results into the general practice system. These functionalities address previously stated requirements for both patients and professionals. The findings of this study will lead to recommendations for a potential cost-effective strategy that can be used for the implementation of IBSM in practice.

This study has been designed for translation of research findings into actual practice. Primarily, the pre-cluster randomization design allows us to study the effect of the different implementation strategies on the number of primary care practices that participate in IBSM. Second, the impact of the different strategies at both professional and patient level on actual implementation (*i.e.*, participation, patient outcomes, and cost-effectiveness). Third, general practices are given a leading role to adapt and internalize IBSM in clinical practice: the project team will only act as facilitators to these practices. Additionally, the influence of research activities on implementation has been incorporated in the design of this study, because these might function as an extra stimulus for both practices and patients to adapt and internalize IBSM during the study period. Therefore, the content of the MS strategy is not only similar to commonly used (non-tailored) implementation strategies, but also the number and amount of research activities are minimized. This is in contrast to the IS and ES strategies, which are specifically designed to address previous identified barriers. These differences give us the opportunity to study how much support is required by general practices for IBSM implementation. Finally, this study evaluates the costs of the implementation from a societal perspective, which is in contrast to other implementation studies [18,48]. Therefore, we will include costs for asthma care from both non-participating practices and non-participating patients.

### Implications

The results of this study will help to determine the most effective way of implementing IBSM. Both the pre-cluster randomization and the inclusion of the IS and ES strategies provide a more detailed view of the implementation process and thereby direction to the focus for future implementation processes in primary care. For example, it may provide information on: the quality and intensity of support professionals need to identify eligible patients; whether it is sufficient to focus only on the start-up period of implementation; and whether continuing support of professionals has additional value in maintaining implementation.

The results of this study can enhance a broad implementation of IBSM in current clinical care, based on a cost-effective strategy. Thereby it can contribute to improved care for patients with asthma. Furthermore, design and results of this trial can contribute to development of effective implementation strategies for self-management initiatives for other chronic diseases.

### Ethical approval

This study has been approved by the Medical Ethics Committee of the Leiden University Medical Centre.

## Competing interests

All authors declare that they have no competing interests. JKS received unrestricted research grants by the Netherlands Asthma Foundation, the Netherlands Organisation for Health Research and Development (ZonMW), Fonds NutsOhra, GlaxoSmithKline NL and study equipment by AstraZeneca NL and Aerocrine, Sweden.

## Authors’ contributions

JB, JG, JS, LB, and MB were involved in the design of the study; JG, JB. JS, and LB drafted the manuscript, which was reviewed by AK, BT, CT, BT, MB and VM. The manuscript has been read and been approved by all authors.
